# Book Reviews

**Published:** 1896-10

**Authors:** 


### BOOK REVIEWS.

A Manual of Clinical Diagnosis by Microscopical and Chemical Methods. For Students, Hospital Physicians, and Practitioners. By Charles E. Simon, M.D., Late Assistant Resident Physician John Hopkins Hospital, Baltimore. In one very handsome octavo volume of 504 pages with 132 engravings, and 10 full-page colored plates. Cloth, $3.50. Lea Brothers & Co., Philadelphia and New York, 1896.

The blood, the various secretions and excretions are all carefully considered in this book from the point of view of diagnosis, and a very important matter it is that physicians should be better acquainted than they generally now are with this subject, in which Simon's work should help to foster an interest, for it is clearly written and arranged, as well as copiously illustrated, and contains a vast amount of information. The publishers have also done their part well, and the result is a work which is heartily to be commended.

---

Diagnostic Urinology. By M. D. Hoge, Jr., M.D., Richmond, Va., Professor of Histology, Pathology, and Urinology University College of Medicine, etc. pp. 81. Price, prepaid by mail, $1.00. To be had from the author.

This is a compact little book, much of which is arranged in tabular form and contains information for students and practitioners necessary in investigating the condition of the urine. It is a useful book to have at hand.

---

Anatomy, Descriptive and Surgical. Henry Gray, F.R.S., Fellow of the Royal College of Surgeons; Lecturer on Anatomy at the St. George's Hospital Medical School. A new edition thoroughly revised by American authorities. From the Thirteenth English Edition. Edited by F. Pickering Pick, F.R.C.S., with 772 illustrations, many of which are new. Lea Bros. & Co., Philadelphia and New York, 1896.

Gray's Anatomy has been the standard for many years. The improvements which have been made in its text and illustrations in the past ten years are wonderful. If the old "Gray" was the best, the latest "Gray" is a double superlative.

This 1896 edition is simply an improvement upon the one of 1893. The sections on the brain, teeth, and purely abdominal viscera have been rewritten. The many excellent old illustrations have been retained and numerous new ones added. The illustrations of the peritoneal relations are especially striking. Cuts of special points in the anatomy add to the value of the book.

Especial attention, as heretofore, is given to the surgical anatomy and the dissections shown are remarkably clear.

A student once said that he could learn more from Gray's Anatomy than from dissecting. This would be true of the average dissector, if he used the edition under consideration. It is Gray's Anatomy vastly improved over the old Gray. M. B. H.

---

A MANUAL OF VENEREAL DISEASES. By James R. Hayden, M.D., Chief Venereal Clinic at the College of Physicians and Surgeons (Columbia University), New York; Professor of Genito-Urinary and Venereal Diseases in the Medical Department of the University of Vermont; Visiting Surgeon to the New York City Hospital. With forty-seven illustrations. Lea Bros. & Co., New York and Philadephia, 1896.

This is a concise manual, describing the symptoms and treatment of gonorrhea, chancroid, and syphilis. As necessary in a manual, the text is very concise and only gives the essentials in symptoms and treatment. The illustrations are chiefly of instruments, and the methods of their employment. The treatment and formula follow Dr. R. W. Taylor. It is a convenient reference book. M. B. H.

---

JACKSON'S READY-REFERENCE HANDBOOK OF SKIN DISEASES. The Ready-Reference Handbook of Diseases of the Skin. By George Thomas Jackson, M.D., Professor of Dermatology, Woman's Medical College of the New York Infirmary and in the University of Vermont; Chief of Clinic and Instructor in Dermatology, College of Physicians and Surgeons, New York. New (2d) Edition. In one 12mo volume of 589 pages, with 69 illustrations and a colored plate. Cloth, $2.75. Philadelphia, Lea Brothers & Co., 1896.

Dr. Jackson is one of the most thoroughly learned of the dermatologists in the literature of skin diseases, as is shown by the text of this book. The matter has been brought down to date, several new diseases receiving notice. The text is extremely brief, as is to be expected in a small work. The alphabetical arrangement of the diseases is followed, as in the first edition. Illustrations are for the most part good. M. B. H.

---

PTOMAINS, LEUCOMAINS, TOXINS, AND ANTITOXINS, OR THE CHEMICAL FACTORS IN THE CAUSATION OF DISEASE. By V. C. Vaughan, Ph.D., M.D., and F. C. Hovy, Sc.D., M.D., of the Uinversity of Michigan. pp. 558. Published by Lea Brothers, Philadelphia.

The popularity of this work is proven by the appearance of a third edition. The importance of chemistry in relation to disease is daily becoming more marked, and a knowledge of this relationship is of value to the practitioner as well as to the public health officer in his investigation into the origin of outbreaks of disease. This book enters thoroughly into the researches which have been made in different countries in connection with food poisons, the bacteria of many of the infectious diseases, and the action upon them of blood serum. It considers the subject of immunity, antitoxins, and general serum therapy, as well as the methods of isolating ptomains and toxins, their chemistry and their resemblance in action to various well known poisons. In these days, when the hopes of the profession for the ultimate cure of some of the more deadly diseases lie in the region traversed by this work, a careful study of it cannot but well repay the reader.

The publishers have done well their part of the work.

---

THE FUNDUS OCULI. By Adams Frost, F.R.C.S., Ophthalmic Surgeon, St. George's Hospital; Surgeon to the Royal Westminster Ophthalmic Hospital, London. Young J. Pendland, Edinburgh and London, and McMillan & Co., New York. Price, $18.00.

Mr. Adam Frost has for years been giving several courses of lectures during the year at St. George's and at the Royal Westminster Ophthalmic Hospitals, on the fundus of the eye in health and disease, illustrated by magic lantern slides. These lecturers are most interesting and instructive, and the present book is an amplification of them. From long acquaintance with a great part of this work before its publication, the writer can bear witness from personal knowledge to the immense amount of care which was expended upon it during its years of preparation. That this care has been expended to full advantage will be admitted by all who use the book, whose text, which contains in a most readable form all information concerning the various appearances of the fundus, and is printed in large type and on thick paper, is illustrated by 107 beautifully colored plates, besides numerous other illustrations of great value. The whole forms a book which must take its place as one of the most valuable, if not the most valuable, which has ever appeared in connection with this subject.

---

PARK'S TREATISE ON SURGERY. A Treatise on Surgery. By American Authors. Edited by Roswell Park, M.D., Professor of Surgery and Clinical Surgery, Medical Department, University of Buffalo, Buffalo, N. Y. In two very handsome octavo volumes, comprising about 1,600 pages, with about 800 engravings, largely original, and about 40 full-page plates in colors and monochrome. Volume I., General Surgery and Surgical Pathology. Volume II., Special Surgery. Price per volume, Cloth, $4.50; Leather, $5.50. *Net.* Lea Brothers & Co., 1896.

The contents of volume I. are divided into five parts, surgical pathological, surgical diseases, principles and methods and minor procedures, injury and repair, and surgical affections of the tissues and tissue-systems. These subjects are fully and carefully dealt with, and remarkably well illustrated. Indeed, the illustrations comprise one of the most noticeable merits of this volume; they are numerous and well executed. Among those which have specially attracted the writer's notice are illustrations dealing with accidents from anesthetics and the test methods for their prevention and relief, a subject to which Dr. Hare, the writer of this section, has given much attention. Each section of this book having been written by a surgeon specially chosen on account of intimate knowledge of the subject allotted to him, it is needless to say that it contains work of only a high order, and that the student and practitioner will find full and accurate information in its pages on the surgical questions with which it deals.

SPECTACLES AND EYE-GLASSES. Their Forms, Mounting and Proper Adjustment. By R. J. Phillips, M.D., Adjunct Professor of Diseases of the Eye, Philadelphia Polyclinic, etc. Second Edition. P. Blakiston, Son & Co., 1012 Walnut Street, Philadelphia. Price, $1.00.

This little book is "intended to supplement studies in refraction, and to give the student that knowledge of the correct placing of the glasses before the eyes, without which the most painstaking measurement of the refraction will frequently fail of practical result." Systematic works on ophthalmology and refraction frequently omit all discussion of the mechanical aspect of glass-fitting and adjustment. The author has succeeded in this book in presenting this subject in a very clear and practical manner. Oculists will find it a useful supplement to their works on ophthalmology. The contents embrace a description of the material and manufacture of lenses, the principles of spectacle fitting, the prescription of frames, the adjustment of spectacles and eye-glasses, the different patterns of spectacles, etc.

---

We have received of Prof. Chas. Marchand the eleventh edition of his book upon his medicinal agents and their numerous uses in the diseases due to micro-organisms. The subject is very thoroughly treated, and various quotations are made from writers.

The book can be obtained of Professor Charles Marchand, No. 28 Prince Street, New York. M. B. H.

---

REMINGTON BROTHERS' NEWSPAPER MANUAL, 1896.

We have received the ninth issue of the Newspaper Manual of Remington Brothers, of Pittsburgh, Pa., and New York, N. Y.

The contents include complete lists of all newspapers in the United States and Canada, with their days of issue, politics and circulations, and properly classified lists of the principal Dailies and Weeklies, and the best Agricultural, Religious, Scientific, and Trade publications and leading Magazines. All the lists are catalogued by towns in alphabetical order, and in the general list the population is given of each town and of the county in which it is located.

The Manual contains a vast quantity of valuable information, concisely arranged, and is handsomely and substantially bound. As a book of reference it must prove invaluable in every business office, as well as to every one doing business as an advertiser.

Sent by Express, prepaid, on receipt of $3.00.

